# Partial Least Squares Regression Can Aid in Detecting Differential Abundance of Multiple Features in Sets of Metagenomic Samples

**DOI:** 10.3389/fgene.2015.00350

**Published:** 2015-12-17

**Authors:** Ondrej Libiger, Nicholas J. Schork

**Affiliations:** ^1^Research and Informatics, MD Revolution Inc.San Diego, CA, USA; ^2^Department of Human Biology, J. Craig Venter InstituteLa Jolla, CA, USA; ^3^Departments of Psychiatry and Family Medicine and Public Health, University of California, San DiegoLa Jolla, CA, USA

**Keywords:** multivariate regression, diversity, abundance, metagenomics, microbiome, statistical power

## Abstract

It is now feasible to examine the composition and diversity of microbial communities (i.e., “microbiomes”) that populate different human organs and orifices using DNA sequencing and related technologies. To explore the potential links between changes in microbial communities and various diseases in the human body, it is essential to test associations involving different species within and across microbiomes, environmental settings and disease states. Although a number of statistical techniques exist for carrying out relevant analyses, it is unclear which of these techniques exhibit the greatest statistical power to detect associations given the complexity of most microbiome datasets. We compared the statistical power of principal component regression, partial least squares regression, regularized regression, distance-based regression, Hill's diversity measures, and a modified test implemented in the popular and widely used microbiome analysis methodology “Metastats” across a wide range of simulated scenarios involving changes in feature abundance between two sets of metagenomic samples. For this purpose, simulation studies were used to change the abundance of microbial species in a real dataset from a published study examining human hands. Each technique was applied to the same data, and its ability to detect the simulated change in abundance was assessed. We hypothesized that a small subset of methods would outperform the rest in terms of the statistical power. Indeed, we found that the Metastats technique modified to accommodate multivariate analysis and partial least squares regression yielded high power under the models and data sets we studied. The statistical power of diversity measure-based tests, distance-based regression and regularized regression was significantly lower. Our results provide insight into powerful analysis strategies that utilize information on species counts from large microbiome data sets exhibiting skewed frequency distributions obtained on a small to moderate number of samples.

## Introduction

Advances in genomic technologies have enabled the exploration of the composition and diversity of microbial communities (“microbiomes”) based on DNA sequencing assays, (Handelsman et al., 1998; Turnbaugh et al., 2007). In recent years, focus has shifted from mainly descriptive studies to analyses that compare the microbiome in different environments or in individuals with and without a disease (Qin et al., 2012). A number of studies found links between changes in microbial communities in the human body and various diseases (Dumas et al., 2006; Turnbaugh et al., 2006; Hartman et al., 2009; Sekirov et al., 2010; Burcelin et al., 2011; Kau et al., 2011; Marchesi et al., 2011; McLoughlin and Mills, 2011; Saulnier et al., 2011).

Such studies require rigorous statistical techniques that test hypotheses regarding differences in microbial composition between sets of samples. However, metagenomic data exhibit several characteristics that complicate relevant analyses via standard statistical methods. Microbial abundance is usually measured on a large number of features (e.g., taxonomic units or genes) in a much smaller number of samples (e.g., patients), leading to a very high dimensionality of resulting datasets. High dimensionality can create general statistical problems such as multicollinearity (Dormann et al., 2013), which can be further exacerbated by the co-occurance of related features (e.g., phylogenetically related taxonomic units) within a sample. Additionally, microbiome species abundance estimates are often highly skewed as only a few very common features are present alongside many rare features, presenting challenges for many standard statistical tests. In addition, many features are likely to be found in only a subset of samples, causing data sparsity. Additional information (“metadata”) describing the samples is also often available, and needs to be appropriately accommodated in the analyses as covariates.

A common approach to testing whether differences exist between the microbiomes of two (or more) sets of samples involves comparing the abundance of *individual* members of taxonomic units or functional categories between the sets of samples using statistical tests for equality of group means or medians (e.g., Rodriguez-Brito et al., 2006; Markowitz et al., 2008; Kristiansson et al., 2009; Schloss et al., 2009; White et al., 2009; Goll et al., 2010; Parks and Beiko, 2010; Lingner et al., 2011; Arndt et al., 2012; Hoffmann et al., 2013). For example, Metastats (White et al., 2009) detects differentially abundant features using *t*-tests and handles sparsely-sampled features via a Fisher's exact test. Association between abundance data and a quantitative phenotype can be tested using e.g., linear regression (e.g., Baran and Halperin, 2012). The main drawback of these methods lies in the fact that they test association of each taxonomic or functional unit separately, so subtle differences in individual abundances that add up to a clinically relevant difference in abundance profiles may remain undetected.

One possibility for detecting a difference between samples' abundance profiles as a whole rather than individual features is comparing the samples' diversity or dissimilarity. Diversity indices can be used to assess a given sample in terms of the richness and evenness of the taxonomic or functional features present within the sample (Hill, 1973; Jost, 2008). Differences between sets of samples can then be assessed by testing the null hypothesis of no difference in the mean of the diversity values associated with one set of samples from the mean value associated with another set (see e.g., Pérez-Cobas et al., 2013; Holler et al., 2014). Alternatively, inferences can be made based on the diversity measured within one sample compared to the diversity of several combined samples, analogously to differentiation measures such as Fst used in population genetics (Wright, 1951; Cockerham, 1973). Contrary to diversity, dissimilarity measures how two samples differ from each other (Jaccard, 1900; Sørensen, 1948; Chao et al., 2005). Sets of samples can then be compared based on the proportion of variation in the dissimilarity values that can be explained by the grouping of samples into predefined sets (Anderson, 2001).

A host of other multivariate statistical techniques can address issues in the analysis of microbial abundance data. For example, La Rosa et al. (2012) introduced a parametric mutlivariate model based on Dirichlet-multinomial distribution that can be used to detect differences between sets of samples. While powerful in some instances, the drawback of this method is that it does not reliably detect differences between infrequent taxa or features. In the example datasets used in our studies, and in many other datasets, such rare taxa account for the majority of taxonomic units. Dinsdale et al. (2013) showcased the use of k-means clustering, classification trees, random forest, multidimensional scaling, linear discriminant analysis, principal component analysis, and canonical discriminant analysis to identify metabolic functions that differ in previously published metagenomes collected in different environments. In light of these surveys and studies, it is important to explore and compare the power and sensitivity of the various methodologies to detect differences between sets of samples using simulated realistic data where the degree and type of difference is known.

We considered the use of several statistical techniques developed in other fields, including principal component regression, partial least squares regression, regularized regression, and distance-based regression, for the analysis of microbiome data and explore their statistical power using simulations that introduce an effect in realistic settings. We also compared the performance of these methods to methods that have been used in the context of comparing microbial communities or have been described in the literature, specifically resampling based statistical test involving a family of Hill's diversity measures described by Pallmann et al. (2012), and a univariate set of tests with multiple testing correction implemented in Metastats (White et al., 2009), which we modified for the purpose of multivariate analyses. Based on our results, we provide concrete guidance as to which methods are most appropriate for the comparison of microbial communities from two sets of samples. Our study can shed light on the utility of methods being considered for use in studies seeking to determine whether people with a certain disease possess a different microbiome compared to healthy individuals.

## Methods

### Data

**Figure 1:**
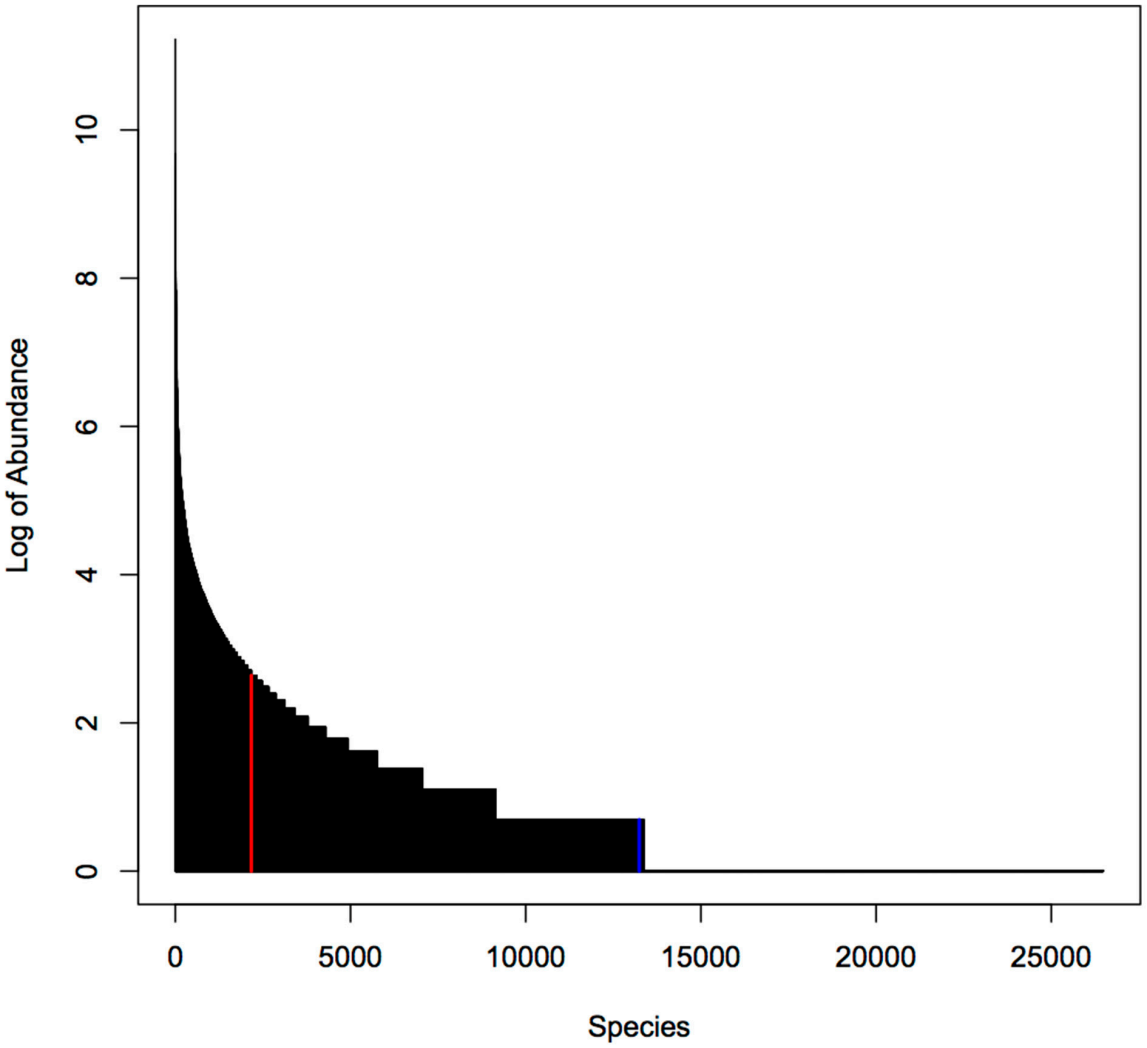
**Distribution of abundance (averaged over 88 samples)**. Red line indicates median abundance, blue line indicates average abundance. Note that y-axis is on a log scale.

For our simulation studies we used published data from a microbiome study examining the palms of human hands (Fierer et al., 2008). This study provided abundances of 48,237 operational taxonomic units (OTUs) for male and female individuals. We used data on 26,482 OTUs with greater than zero abundance in 88 male individuals. As shown in Figure 1, the distribution of OTU abundance is highly skewed: there are a few highly abundant OTUs and many rare OTUs. Quartiles of abundance (averaged across the 88 samples) are 0.011, 0.011, 0.023, 0.045, and 850.44. Mean abundance (averaged across the 88 samples) is 0.16 at the 2169th OTU sorted from the highest to lowest average abundance.

### Power analysis

We used previously published data on operational taxonomic unit abundance in 88 human hands. These 88 samples were randomly distributed into two groups (i.e., making up the cases and controls) 100 times. Then we introduced, *in silico*, changes of varying magnitude to the abundance profile associated with the samples in the case pool (see disease model description in Methods for details). Subsequently, abundance profiles were normalized so that each abundance value reflected the proportion of the observations associated with each taxonomic unit to all observations. Thus, abundance of 0.1 reflected an absolute abundance equal to 10% of the total abundance across all taxonomic units.

The sensitivity of detecting changes (or “power”—although we realize there are very nuanced uses of the term power by theoretical statisticians) of each technique was measured as the number of times (out of 100) that a particular technique detected change in the abundance profile between the case and control samples. For example, if one technique detected the change 60 times out of 100, while another technique detected the change 80 times out of 100, the second technique was deemed to exhibit better statistical properties in terms of the sensitivity/power to detect an effect.

**Figure 2:**
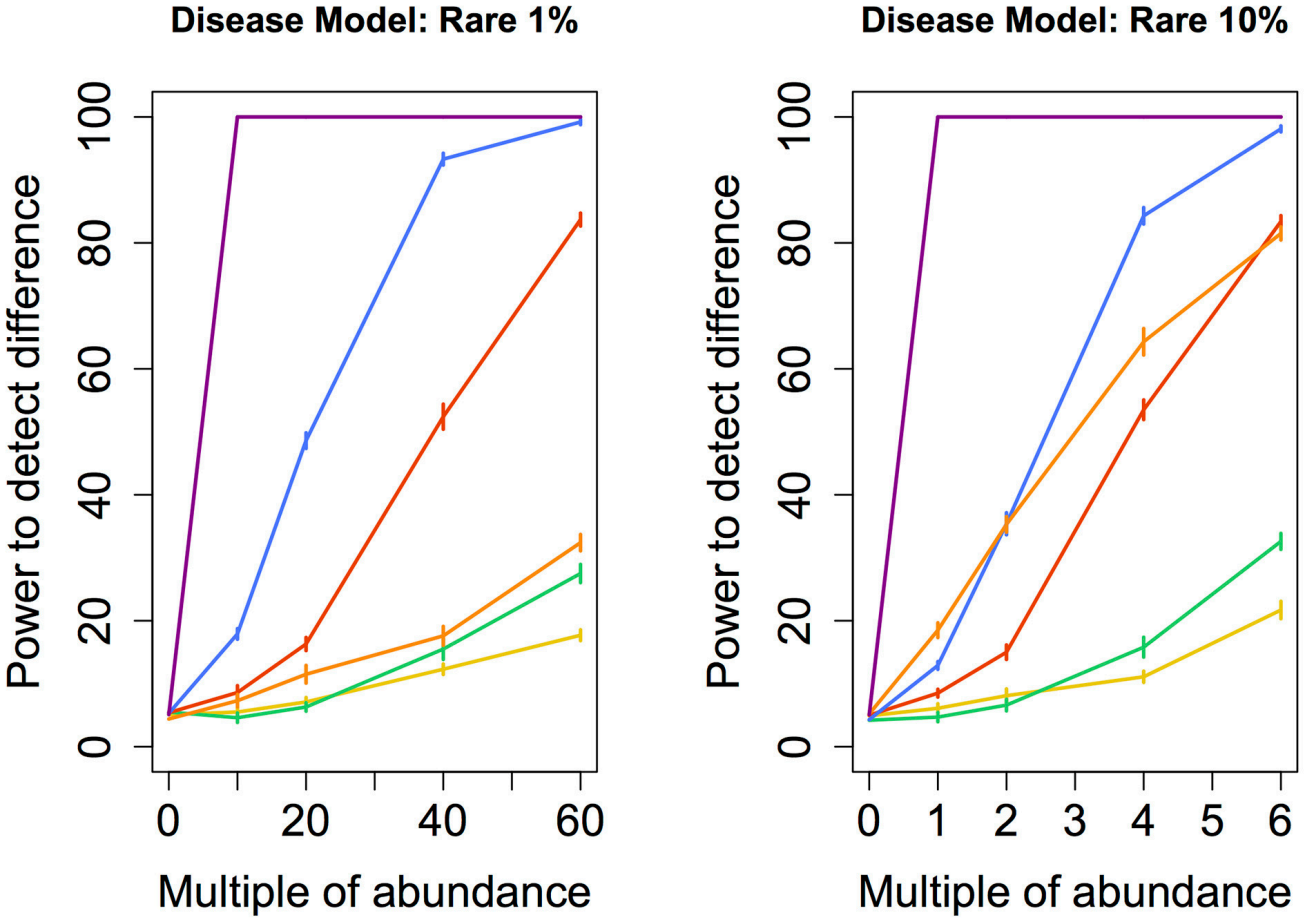
**Rare disease model: power comparison of the best performing statistical techniques from each category**. Bars represent standard errors of the mean. [Purple: modified metastats; Blue: Partial least squares regression (50 components); Red: principal components regression (50 components); Orange: diversity; Green: distance-based regression (Manhattan distance); Yellow: regularized regression (Lasso)].

**Figure 3:**
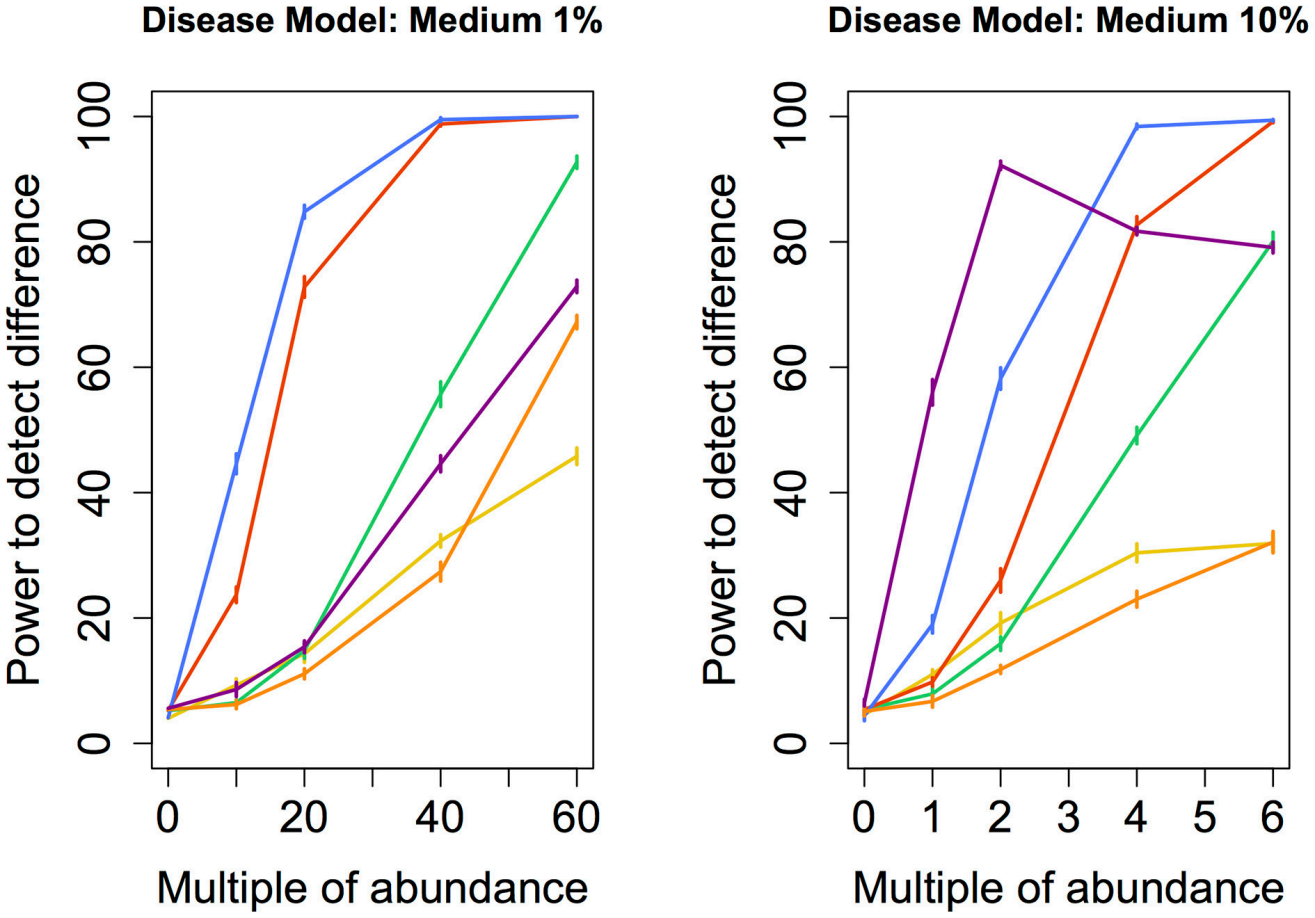
**Medium disease model: power comparison of the best performing statistical techniques from each category**. Bars represent standard errors of the mean. [Purple: modified metastats; Blue: Partial least squares regression (1%-10 components, 10%-50 components); Red: principal components regression (50 components); Orange: diversity; Green: distance-based regression (1%-Manhattan distance, 10%-Minkowski distance with *p* = 0.5); Yellow: regularized regression (1%-Lasso, 10%-Ridge regression)].

**Figure 4:**
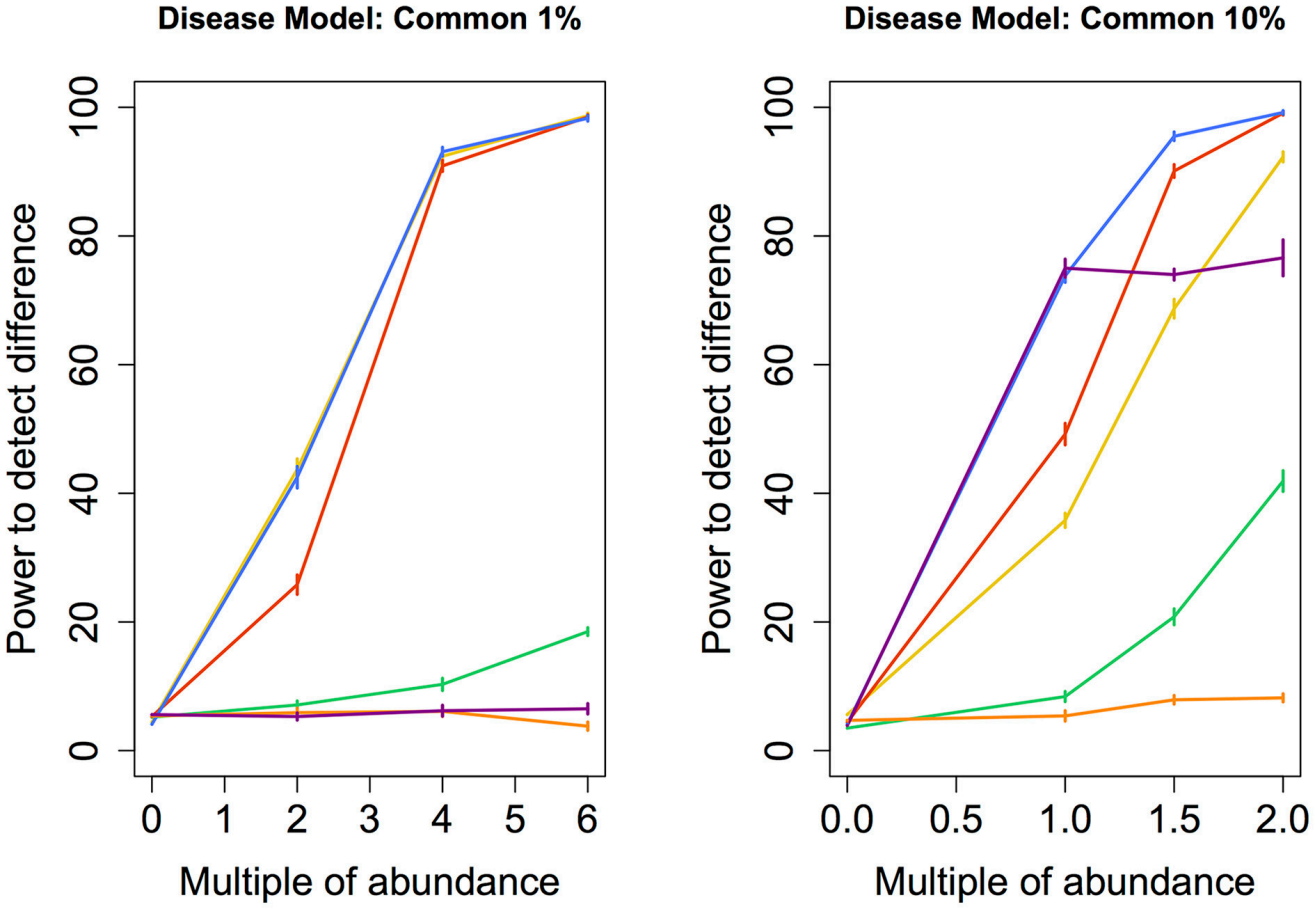
**Common disease model: power comparison of the best performing statistical techniques from each category**. Bars represent standard errors of the mean. [Purple: modified metastats; Blue: Partial least squares regression (10 components); Red: principal components regression (50 components); Orange: diversity; Green: distance-based regression (Manhattan distance); Yellow: regularized regression (Lasso)].

**Figure 5:**
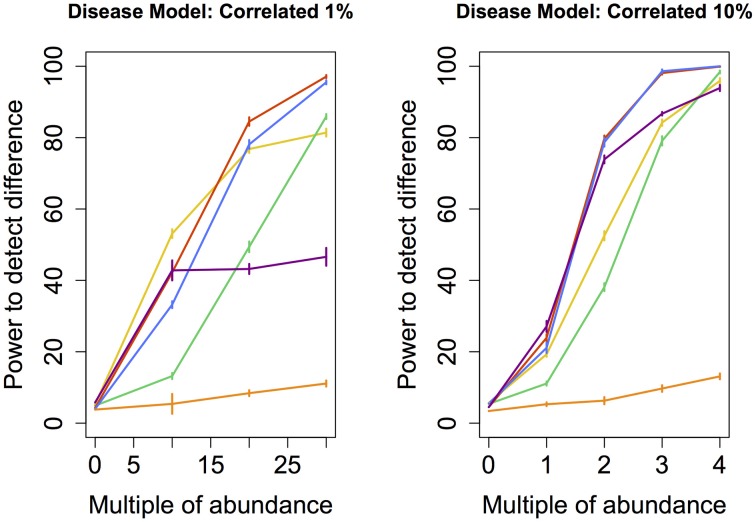
**Correlated disease model: power comparison of the best performing statistical techniques from each category**. Bars represent standard errors of the mean. [Purple: modified metastats; Blue: Partial least squares regression (all components); Red: principal components regression (all components); Orange: diversity; Green: distance-based regression (Manhattan distance); Yellow: regularized regression (Lasso)].

To determine whether the observed difference in sensitivity or power between different techniques was itself statistically significant, we repeated the entire assessment of the power of each technique (i.e., the random grouping of samples into case and control pools carried out hundred times) ten times. This step allowed us to calculate standard errors associated with the empirically-derived power. Ten consecutive estimates of statistical power for each technique was enough to obtain standard errors small enough to provide evidence of statistically significant differences in sensitivity or power between the techniques. These errors (depicted in Figures 2–5) indicate that the differences in sensitivity or power between the various statistical techniques are in fact statistically significant.

### Disease models

We artificially increased the abundance of rare, medium-abundant and common sets of species as well as a set of species with correlated abundance in a group of “case” samples. We then tested the ability of a number of multivariate methods to detect a difference in abundance profiles between the “case” samples and an equal number of “control” samples. For easier comparison, we increased the abundance of species in each case so that the original cumulative abundance (i.e., the abundance among the control group) of this set of species amounted to 1 and 10% of the total abundance associated with all species. In total, we employed eight disease models listed below in our simulation study.

- *Rare 10% (1%)*: the abundance of 21,712 (3832) units with the least average abundance was increased by 1–5 times (1–40 times).
- *Medium 10% (1%)*: the abundance of 2315 (261) units with average abundances centered around the mean average abundance (0.164) was increased 1–5 times (1–40 times).
- *Common 10% (1%)*: the abundance of 109 (11) units with average abundances centered around the abundance of the hundredth most abundant unit (3.7) was increased 1–5 times (1–40 times).
- *Correlated 10% (1%)*: the abundance of 1660 (238) units with average abundances most highly correlated with the abundance of the hundredth most abundant OTU (3.7) was increased 1–5 times (1–40 times).

### Statistical techniques

We classified the techniques into six broad categories, which are described below. Within each of these categories several related methods were used with different parameters. We describe each of these in isolation below.

#### Modified metastats

Metastats is a publicly available analysis software developed by White et al. (2009). It detects differentially abundant features using *t*-tests and handles sparsely-sampled features via a Fisher's exact test. Significance is determined using resampling and false discovery rate procedure. This technique was designed for univariate analyses, i.e., serial tests of differences in abundance in a single taxonomic unit or feature. We modified this technique for multivariate analyses by using the number of individual features found to significantly differ as a “global” test statistic. We then used resampling described below to derive the null distribution of this “global” test statistic using the original dataset. This allowed us to assess the likelihood of observing the data generated in the resampling procedure under the assumption of no increase in abundance in the case samples. Based on this null distribution, we derived a *p*-value associated with each time Metastats was applied to a set of control and case samples. For example, if only 1% of statistics under the null distribution were higher than the observed test statistic, the *p*-value associated with the overall test equaled 0.01. Whenever the *p*-value was lower than 0.05, the test was considered to yield a statistically significant result. The statistical power was determined as the number of statistically significant results obtained from the 100 replications under the alternative hypothesis.

#### Regularized regression

Regularized regression models are a class of linear models with a constrained sum of regression coefficients. In regularized regression, models with extreme coefficient values are penalized. This can prevent overfitting and produce a sparse model. These techniques include the popular lasso, ridge regression and elastic net methods, which are widely used in biomedical sciences (e.g., Malo et al., 2008; Li et al., 2011) to analyze high-dimensional, collinear data (Dormann et al., 2013). In addition, lasso is suitable for classification, prediction as well as variable selection purposes (Friedman et al., 2010). We fit a logistic model via penalized maximum likelihood employing a lasso, ridge regression and elastic net penalties. Ten-fold cross-validation was used to choose the lambda penalty. A quantile of the chi-square distribution associated with the difference between model and null deviance with degrees of freedom reflecting the number of nonzero coefficients in the model was used as the test statistic describing the fit of the lasso and elastic net model. Only the difference between model and null deviance was used in the case of ridge regression due to the large number of degrees of freedom.

#### Multiple distance-based multivariate regression (MDMR)

MDMR is rooted in linear models. It essentially seeks to predict or explain variations in a distance or dissimilarity matrix, enabling the quantification of the amount of variation contained within the matrix to predefined grouping of the variables (McArdle and Anderson, 2001; Zapala and Schork, 2012). We constructed distance matrices from the abundance profiles using Minkowski distance measure with parameters equal to 0.25, 0.5, 1 (i.e., the Manhattan distance), 2 (i.e., Euclidean distance), and 4. The pseudo-F statistic was used as the test statistic.

#### Principal component regression (PCR)

Principal components are projections of observed data in the vector space formed by othogonal eigenvectors. Multi-dimensional datasets are often analyzed using a subset of principal components associated with the largest eigenvalues. This approach reduces the dimensionality of the original data, while retaining maximum variance. Comparative metagenomic studies often use principal component analysis and other related ordination techniques to reduce the dimensionality of the data and to visualize information based on some measure of the samples' dissimilarity (e.g., Ramette, 2007; Brulc et al., 2009; Willner et al., 2009; Kuczynski et al., 2010). Many such tools exist (e.g., Caporaso et al., 2010; Goll et al., 2010; Lingner et al., 2011; Arndt et al., 2012). However, principal component analysis can also be combined with linear modeling to carry out a logistic regression analysis with case/control grouping as the response variable, and a set of suitable eigenvectors as predictors (Chatterjee and Price, 1981; Draper and Smith, 1981). PCR can be used to reduce the high dimensionality of the data while at the same time statistically test hypotheses regarding the samples' grouping into predefined sets. In addition to including all principal components of the abundance matrix in the linear model, we also tested models with 5, 10, and 50 top principal components. Coefficient of multiple determination (R2) was estimated using ten-fold crossvalidation and used as a test statistic describing the overall model fit (Mevik and Cederkvist, 2004).

#### Partial least squares regression (PLSR)

A technique related to principal components regression is PLSR, which prioritizes principal components in the model that covary with the response rather than their variance as is the case in PCR (Wold et al., 1969; Garthwaite, 1994). Similar to PCR, we included all principal components in the linear model, as well as sets of 5, 10, and 50 top principal components. Thus, in PLSR, different sets of components were included in the regression model compared to PCR. Coefficient of multiple determination (R2) was estimated using ten-fold crossvalidation and used as a test statistic describing the overall model fit (Mevik and Cederkvist, 2004).

#### Diversity measures

Changes in abundance between two samples can also be done by comparing the diversity of the features within one sample with that of another. Diversity is most often assessed in terms of the richness and evenness of features within a given sample. However, a large number of diversity measures exist that differ in their sensititvity to differences in abundance of rare and frequent species. This makes it difficult to always choose an appropriate diversity measure for the data at hand, because the nature of the differences is often not known before the analysis is carried out. To overcome this problem (Pallmann et al., 2012) devised a method based on Hill's diversity measures (Hill, 1973), which simultaneously tests a family of diversity measures, and refines parameters based on the specific dataset that is being analyzed. We used the multiplicity adjusted *p*-value as the overal test statictic.

## Derivation of the null distribution of test statistics

Since the theoretical distributions of the tests statistics, including distributions under the null hypothesis, generated by some of the techniques are unknown, we carried out a resampling study to obtain empirical distributions under the null hypothesis of no difference between the two sets of samples. These empirical distributions were then used to derive a significance threshold of the test statistics associated with a 0.05 alpha level. A null hypothesis by definition assumes that no effect/difference is present. In our case, this is true only in the original data, before any changes in abundance profiles are introduced by our procedure. For this reason, we use the original dataset to determine the distribution of the test statistic under the null hypothesis since there would be no reason (outside of purely random allocation) that arbitrarily or randomly assigning case and control status to the original data set would induce differences between the cases and controls, as would be the case if we used purely simulated data in this manner.

Specifically, we sampled 44 abundance profiles (out of the 88 available) 100,000 times, and applied each statistical technique to compare the abundance profiles associated with the sampled set against the remaining 44 profiles. The significance threshold was set to equal the test statistic value such that exactly 500 tests (out of 100,000, i.e., 5%) yielded values greater or equal to that threshold value. This procedure thus allowed us to control the type I error rate at 5%, i.e., set the alpha level to 0.05. The subsequent resampling procedure outlined in “Power analysis” section generated 100 test statistics for each technique and “disease model.” The number of times that this test statistic crossed the threshold derived from the null distribution determined each technique's statistical power to detect differential abundance.

## Results

We applied a set of statistical techniques to the entire abundance profiles, i.e., abundance information associated with every species (We use the term species as a shorthand for operational taxonomic units, which constitute the features in this study). No information about which particular species' abundance had been augmented was used as input in the analyses. We found that only some of the techniques were able to detect a differential abundance between two groups of samples. The most powerful methods achieved 80% power when the abundances of the smaller set of species increased approximately by a factor of 20. The abundance of the larger set of species needed to increase between 3 and 4 times before the difference could be detected with 80% power.

When abundance was increased only for infrequent species (models *Rare 1 and 10%*), testing for univariate differences using *t*-test and Fisher's exact test (as implemented in Metastats), and comparing the total number of significantly different species to an empirical distribution generated when no increase in abundance was introduced, yielded by far the highest power for both the smaller set as well as the larger set of species. However, modified Metastats is also more computationally demanding. The statistical power of most methods was quite similar when sets of infrequent species were augmented, with the exception of diversity measures, which exhibited significantly higher power in analyses involving only the smaller set of species.

We also tested the power to detect a difference in abundance profiles when the abundance of species occuring at medium frequency in the samples were augmented. Partial least squares regression yielded highest power, followed by the principal component regression. Although modified Metastats performed quite well under the 10% model, it's power did not increase monotonously with increasing difference in abundance, and thus cannot be recommended.

Under the *Common* models, abundance of only a very small number of species was increased (11 and 109 resp.). Similar to *Medium* models, partial least squares regression and principal components regression performed better than the modified Metastats method. Newly, regularized regression technique Lasso perfromed very well in this model, especially in the case when the abundance of fewer species had been increased.

Perhaps the most realistic model that we considered involves increasing the abundance of species whose original abundance levels are highly correlated (*Correlated* 1 and 10%) because phylogenetically related species are likely to change abundance in concert with one another. However, a high correlation in abundance can only be determined between species that are somewhat prevalent in the sample. For this reason, none of the species whose abundance was augmented in these models were rare. Perhaps not surprisingly, the results are similar to those observed under *Common* and *Medium* models, with the notable difference of principal components regression outperforming partial least squares regression.

Another set of issues in the analysis of microbiome regards the choice of parameters for techniques within each class of methods. In regularized regression, lasso exhibited higher power than ridge regression (except under Medium 10% model, when ridge regression performed slightly better) and elastic net. In distance-based regression, Manhattan distance (i.e., Minkowski distance with *p* = 1) performed best in most cases. The exception was *Medium 10%* model, in which distance-based regression based on Minkowski distance with *p* = 0.5 exhibited higher power (*p* < 0.05) than the other tested dissimilarity measures. Principal components regression achieved the highest power when 50 (rather than 5, 10, or all) top principal components were included under all models except in *Correlated* models, when all components yielded higher power (*p* < 0.05). Partial least squares regression exhibited the highest power when 50 components were included under *Rare* and *Medium 10%* models, 10 components under *Medium 1%* and *Common* models, and all components under *Correlated* models. These results suggest a general recommendation to include no fewer than 10 top components in principal component regression and at least 5 components in partial least squares regression.

In conclusion, two of the methods, Metastats modified for multivariate analysis and partial least squares regression, yielded high power under all studied models to detect a difference in abundance. The statistical power of diversity measures, distance-based regression and regularized regression was significantly lower.

## Discussion

A number of bioinformatic challenges must be met before the statistical analyses described here can be carried out. These involve sampling, sequencing, assembly, gene calling, assessing diversity and functional annotation. Wooley et al. (2010) provide an excellent review of relevant methodologies. Many online tools exist that address most of these issues (Markowitz et al., 2008; Meyer et al., 2008; Schloss et al., 2009; Caporaso et al., 2010; Goll et al., 2010; Lingner et al., 2011). We explored the problem of comparing sets of metagenomic samples based on feature counts. Features can represent individual taxa, genera, or phyla, but also other “countable” features such as genes, proteins, functional categories, etc. Our approach is thus very general; it can be used to investigate functional diversity, taxonomic composition as well as rank abundance distribution of sets of metagenomic samples, and the count information can be obtained from ribotypes as well as other metagenomic approaches. In addition, the results of this study are applicable to a wide range of endeavors outside of metagenomics that utilize information on counts associated with large number of features observed in a smaller number of samples when the distribution of these counts is highly skewed.

Most comparative metagenomic studies investigate the following two questions: (1) whether sets of samples differ in terms of microbial abundance; and (2) which specific taxonomic units or other features differ when their abundance is compared between sets of samples. Only if the answer to the first question is positive, one would proceed to address the second question. In this article, we aimed to provide a statistical guidance in answering the first question; i.e., determining whether two sets of samples collected from two different microbial communities differ in terms of feature abundance. For this purpose, we examined several techniques that are all readily available and relatively easy to use. All techniques described in this article are implemented in the R statistical computing environment, and, except for Metastats, can incorporate additional important information such as age, sex, etc. as covariates in the model. Although some techniques described here (e.g., the lasso) could also be used to explore the second question, generally, a different set of methods designed specifically for the problem of “variable selection” would be more appropriate.

By leveraging permutation-based significance tests (i.e., *p*-values) we could alleviate assumptions about error distributions of the statistics. While these methods are less assumptions-laden, and can be used with many different kinds of data, they are also generally more computationally intensive due to the need of incorporating the resampling procedure. An additional limitation is that permutation-based methods often assume that the dispersion within the sets of samples is equal. However, overdispersion can occur in various situations, for example when the sets of samples come from very different environments or when cases and controls are compared for a disease that significantly affects the microbial diversity. When severe overdispersion is present, the rate of type I error may increase. It is therefore important to test for the presence of overdispersion in the data and several tests for this purpose exist (Anderson, 2006; La Rosa et al., 2012). In case of a positive result, care should be taken to obtain the appropriate null distribution.

One of the more surprising results of our study was the low power exhibited by techniques based on comparing sample diversity, since these methods are used quite frequently in this context. One reason that may explain this finding is that their power diminishes when the majority of species occur in both sets of samples, albeit at varying relative abundance. In situations when the taxonomic composition of the samples is substantially different, the power of diversity measures may be higher.

Principal components regression, partial least squares regression, and regularized regression are multivariate techniques that are designed to detect subtle shifts in abundance implicating multiple features more easily than a series of univariate tests, especially when there is a complex correlation structure among the features. This could explain why these multivariate techniques outperformed Metastats in *Medium* and *Common* disease models, where the abundance of mutually correlated features is increased. Conversely, in certain *Rare* disease models, which involve features with largely uncorrelated abundances, Metastats exhibited higher statistical power using information from a series of powerful univariate Fisher's exact tests. Interestingly, the statistical power of Metastats did not always increase with increasing effect size (e.g., medium 10% disease model). It is possible that when the abundance of a set of features was increased beyond a certain threshold, Metastats detected all those features as significantly different. Upon increasing the abundance of these features further, no gain in overall statistical power could be obtained.

In our study, we fitted PCR and PLSR models that contained 5, 10, or 50 top principal components as well as models that included all components. Omitting principal components that are associated with small eigenvalues is a commonly used approach intended to prevent over-fitting and reduce noise. Using all principal components was intended to create new variables that better reflect the natural co-occurence of certain species in microbial communities by exploiting the correlations among their observed abundance, rather than reduce the dimensionality of the data. Fitted models that yielded the maximum power for each disease model were then chosen to represent each technique in the power comparison summarized in Figures 2–5. We found that statistical power was dependent on the number of components included in the model. In addition, regression models with different numbers of top components yielded the highest power under various disease models in our study. This finding validated the need for comparing multiple models with varying numbers of top principal components when PCR or PLSR are employed. Alternative approaches that may further improve the observed statistical power include using the Tracy-Widom statistic (see e.g., Patterson et al., 2006) to determine the number of principal components to retain in the model, including only principal components with their associated eigenvalues greater than 1.0, varying the number of components in the model dynamically based on the cumulative amount of variance that they explain, or employing cross-validation studies to determine the optimal number of components to include. However, some of these approaches are associated with additional computational demands.

Fine-tuning the parameter settings in some of the presented techniques may lead to increased statistical power. However, our objective was to compare these techniques as they would be used by researchers that don't necessarily possess specialized expertise in these methodologies. Similarly, increasing the sample size would likely increase the power of each method to detect changes in abundance. However, our goal was to compare the power of these techniques when applied to the same dataset rather than fully characterize the power of each method as a function of varying sample size. The strength of this study lies in using a real dataset with realistic correlation structure among features based on e.g., phylogenetic relationships. Such a realistic correlation structure and skewness of the data would be difficult to simulate without using a real dataset.

### Conflict of interest statement

The authors declare that the research was conducted in the absence of any commercial or financial relationships that could be construed as a potential conflict of interest.
